# Navigating the plastic crisis: How Commitments Can Lead to Polycentric Governance

**DOI:** 10.1038/s44319-026-00784-7

**Published:** 2026-04-24

**Authors:** Matthias Braun, Anna-Katharina Hornidge

**Affiliations:** 1 Department of Social Ethics, University of Bonn, Bonn, Germany; 2 German Institute of Development and Sustainability (IDOS) & Institute of Sociology, University of Bonn, Bonn, Germany

**Keywords:** Economics, Law & Politics, Evolution & Ecology

## Abstract

The failure of negotiations to address plastic pollution and its impact on public health and the environment reflects deeper geopolitical fragmentation and a crisis of multilateralism. It highlights the need to strengthen shared commitments across affective, normative, interest-based, and institutional dimensions to enable global action.

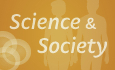

On August 15 last year, after 10 days of intense formal and informal talks in Geneva, Switzerland, UN negotiations to develop an internationally binding instrument to curb plastic pollution failed to reach an agreement. The differences between UN member states who supported a treaty to limit plastic pollution and many oil-producing countries turned out to be too large. The failure of the Geneva meeting, held under the auspices of the United Nations Environment Assembly (UNEA), demonstrates that global environmental governance is undergoing a structural transformation driven by the interplay of biosphere risks, economic transitions, technological competition, and geopolitical fragmentation (Editorial, 2025). It also illustrates the widening gap between the scientific understanding of planetary-scale environmental risks and the institutional mechanisms for addressing these on a global scale (Dauvergne and Allan, 2025).

Plastic contamination represents a persistent public-health and environmental risk rather than merely an industrial-waste problem. Growing evidence from epidemiological, biomedical, and environmental research indicates that plastic waste accumulation and the ensuing microplastics are associated with long-term health risks for humans and for wildlife (Landrigan et al, 2025). At the same time, the scale of the problem urgently demands solutions to reduce the amount of plastic waste that ends up in the environment. Global plastic production has increased from approximately 2 megatons in 1950 to more than 470 megatons in 2022, while global recycling capacity is less than 10%. This demonstrates a systemic imbalance between current linear production models, where plastic ends up as waste, and the mechanisms required for establishing a circular economy based on efficient recycling (ten Hietbrink et al, 2025).

“Plastic contamination represents a persistent public-health and environmental risk rather than merely an industrial-waste problem.”

Despite the scientific evidence and sustained advocacy from civil society, the negotiation processes in Geneva failed to achieve binding regulatory measures. More than a thousand civil-society organizations, scientific networks, and policy advocacy groups called for ambitious treaty commitments. The resulting failure was not merely a diplomatic setback, but it also signals a deeper structural fragility within the fabric of multilateral cooperation (Müller and De Man, 2025).

Against this background, the pressing question is not only who is to blame or how UNEA might proceed in the future. A more fundamental question is: how can the international community still agree on joint action and binding political decisions, given the increasing geopolitical fragmentation? Two overarching developments determine and shape the current impasse. First, highly dynamic and overlapping global crises—including climate change, biodiversity loss, pandemics, economic volatility, and armed conflicts—generate constant pressure on governance systems (Canuto and Saraiva, 2025). Second, geopolitical shifts are weakening the previous model of rules-based multilateral global governance. The major powers—notably the USA and China—show less interest in multinational agreements and treaties that could affect their political and economic interests; Russia has been largely isolated since its invasion of Ukraine; and South America and Southeast Asia have aligned their interests and formed economic and political blocs, such as ASEAN or MERCOSUR.

“A more fundamental question is: how can the international community still agree on joint action and binding political decisions given the increasing geopolitical fragmentation?”

Together, these developments decrease the ability of international institutions to act globally and forge a global consensus on urgent problems and their solutions. Moreover, confidence in the UN to deliver stable solutions has been shaken, despite their historical success to achieve international agreements, such as the Agreement on Marine Biological Diversity beyond National Jurisdiction (BBNJ) or the Kunming-Montreal Global Biodiversity Framework. Reform processes within the UN under the label UN80, initiated by Secretary-General António Guterres, aim to increase agility and efficiency. Yet, they also occur in a climate of declining trust and diminishing global acceptance of Western leadership claims, while leadership offers from the South or Asia are equally contested. The result is not simply institutional fatigue but a broader crisis of confidence in multilateralism itself.

## Commitment as an analytical lens

We argue that overcoming this impasse requires a deliberate shift in perspective. Instead of focusing exclusively on institutions or geopolitical power balances, greater attention should be paid to the underlying commitments of international treaties that build on scientific knowledge and shared promises and expectations. Even in highly adversarial social structures, strong shared commitments can still exist. Conversely, without credible commitments, collective action on planetary crises becomes impossible (North, 1993).

“[…] greater attention should be paid to the underlying commitments of international treaties that build on scientific knowledge, and shared promises and expectations.”

The failure of the plastic treaty negotiations thus signals more than technical or political disagreements. It reveals the fragility of the social fabric of commonality—the erosion of shared narratives and the weakening of commitments to the global common good. To address this, we propose analyzing multilateral governance through four interrelated dimensions of commitment: affective, normative, interest-mediated, and institutional.

## The affective dimension of commitments

Commitments possess an affective dimension. They are created through emotional bonds and a sense of shared belonging and can stabilize cooperation, particularly under institutional uncertainty. Decision-making research suggests that actors behave differently when they perceive themselves as embedded within a larger narrative. Commitments based on a sense of shared belonging may not guarantee optimal outcomes, but they can create a foundation that sustains cooperation and fosters a sense of responsibility (Johnson et al, 2023).

The founding narrative of the UN illustrates this. The 1945 Charter’s opening words—“We the peoples of the United Nations determined to save succeeding generations […]”—articulated a powerful collective narrative. It linked peace, human rights, justice, and social progress into a coherent vision. In the aftermath of a global war, this narrative carried immense emotional force and underpinned the construction of a rules-based international community.

The failure of the plastics negotiations can be interpreted as evidence that this narrative no longer commands the same emotional authority. What once served as self-evident common ground is now contested. Actors operate with different historical memories, developmental, and geopolitical priorities. Particularly states outside the “Old West”, harbor skepticism about assumptions that earlier agreements automatically define present-day obligations.

Addressing the affective dimension, therefore, requires careful analysis of the narratives underlying particular interests. Where do national states locate environmental protection within their broader development? How are economic dependencies on fossil fuels framed within national identity and sovereignty? Emotional responses often indicate precisely when commitments are perceived as non-binding or unjust. If multilateral negotiations are to regain traction, they must reconstruct a shared narrative capable of integrating planetary risk, economic transformation, and geopolitical diversity. This does not imply homogenization but rather the deliberate articulation of overlapping stories that make cooperation more plausible by adding shared, emotionally driven commitments.

## The normative dimension: justifying rules under fragmentation

Commitments also possess a normative dimension. Individuals and institutions adhere to rules not only because of external pressure but because they ultimately regard them as justified. Formal rules and regulations—such as those negotiated within UNEA—represent a layer of governance. They require complementary informal conventions and shared understandings of fairness and legitimacy. When formal rules diverge from informal normative expectations, instability results. The assumption that greater competition among policy proposals will automatically generate institutional innovation overlooks the deeper need for agreeing that these proposals are eventually justified (Northrup, 2014).

“Individuals and institutions adhere to rules not only because of external pressure but because they ultimately regard them as justified.”

During the plastics treaty negotiations, the dynamic often resembled an “all-in game,” characterized by strategic positioning and mutual accusations rather than joint exploration of normative common ground. Re-grounding commitment, therefore, requires explicit deliberation on the reasons for agreeing to binding regulation. What principles justify restrictions on plastic production? Intergenerational justice? Public health? Preservation of biodiversity? Developmental equity? Without shared normative reasoning, procedural adjustments remain superficial.

Moreover, geopolitical fragmentation complicates agreeing on normative consensus. Military and economic power used to be structured primarily along the West-East axis, yet the global order is shifting toward multipolarity. Actors such as China or the EU increasingly articulate distinct normative frameworks. Normative work must therefore occur within a plural environment, seeking overlapping justifications rather than assuming a uniform ideology.

## The interest-mediated dimension: individual and joint commitments in a multipolar context

A third dimension concerns the relationship between individual commitments rooted in vested interests and joint commitments oriented towards the global good. International negotiations are shaped by economic dependencies, industrial structures, and domestic political constraints. Procedural rules attempt to manage asymmetries and ensure transparency and fairness. However, such procedures also compel actors to justify their particular commitments in relation to collective objectives. In the context of plastic reduction, reuse, and recycling, this means explaining how national economic strategies align with the mitigation of planetary risks.

The failed negotiations reveal that individual objectives could no longer be subordinated to a higher shared commitment. Development-oriented and oil-producing nations emphasized economic growth and poverty reduction over mitigating environmental impacts. Resource exporters defended revenue stability. Industrialized states promoted ambitious environmental standards but lacked credibility due to their history of harmful emissions and consumption.

In a cooperative multipolar world, joint commitment cannot be assumed; it must be constructed. Regional and plurilateral formats gain more relevance in this environment. The Shanghai Cooperation Organization demonstrates how Eurasian states achieve coordination outside Western-led structures. The G20 and the G7 continue to enable negotiations between some of the most influential economic powers, but they are currently challenged by substantial political differences among some of their member states. Mercosur, by contrast, illustrates how regional integration can enhance bargaining power and articulate collective economic positions—including negotiations with the EU. A focus on commitments helps to evaluate individual commitments. By doing so, rather than imposing joint commitments top down in a global multipolar context, they can instead be built bottom-up in bilateral and smaller contexts (Table 1).

**Table 1:** Commitments and their implications for navigating a plastic treaty.

Dimension of commitment	Core meaning	Key challenges in the plastics context	Governance implications	Implications for a plastic treaty
**Affective dimension**	Emotional bonds and shared narratives that create belonging and stabilize cooperation	Erosion of the founding narrative of the UN; different historical experiences and geopolitical interests	Reconstruct overlapping narratives integrating planetary risk, development, and geopolitical diversity	The treaty process should rebuild a shared story linking plastic pollution to health, economic transition, and intergenerational responsibility; negotiations should explicitly acknowledge different historical experiences and development pathways
**Normative dimension**	Perceived legitimacy and justification of rules	Strategic “all-in” positioning within UNEA; lack of shared justificatory principles (justice, health, biodiversity, equity)	Develop overlapping normative justifications in a multipolar environment	Treaty provisions must clearly articulate their normative basis – public health, biodiversity preservation, development equity, and so on—procedural fairness and inclusive deliberation as essential for gaining legitimacy
**Interest-mediated dimension**	Relationship between national vested interests and collective global commitments	Growth priorities, revenue stability, credibility gaps; regional coordination through bodies such as the Shanghai Cooperation Organization or Mercosur	Construct joint commitments bottom-up; strengthen regional and plurilateral cooperation	A treaty should allow differentiated pathways and transitional arrangements; regional and plurilateral initiatives can serve as building blocks toward broader multilateral convergence
**Institutional dimension (enforceability)**	Stability and credibility through binding institutions and accountability mechanisms	Fragility of regulatory frameworks under geopolitical pressure; reform debates such as UN80	Strengthen enforceability, transparency, and procedural fairness to rebuild trust	The treaty must include monitoring, reporting, and accountability mechanisms; commitments should be legally binding where possible and protected from short-term political shifts

## The dimension of enforceability and regulatory stability

Finally, credible commitments depend on institutions capable of enforcing adherence to rules and regulations. Promises that can be broken without consequences do not generate trust in institutions. Maybe this is the most important point about commitments here: Even if many people are willing to grant trust in political international institutions, once trust is lost, it is extremely hard to regain. At this point, a clear and committed agenda and institutional leadership have the potential to stabilize long-term expectations and protect agreements from short-term political fluctuations.

Against this background, the UNEA illustrates the fragility of international regulatory frameworks under geopolitical pressure. Yet, transparent and uniform legal structures remain indispensable to maintain or even gain trust in institutions. A prominent example is the Montreal Protocol, negotiated on the basis of robust scientific evidence linking the use of chlorofluorocarbons to stratospheric ozone depletion and related health risks. The strong scientific consensus helped establish a shared problem definition and strengthened both political commitment and public acceptance of regulatory measures. Scientific assessments thus reduced uncertainty among negotiating states and facilitated agreement. Similar dynamics can be observed in regional governance arrangements in the Arctic, where environmental monitoring and ecological assessments have informed cooperative frameworks coordinated through the Arctic Council, even with non-Arctic countries such as Germany or Singapore among others as observers.

Accountability mechanisms are therefore essential components of commitment stability. Reform initiatives, such as UN80, aim to address questions of efficiency and functionality. However, functionality alone does not create credibility. Institutional reform must strengthen enforceability, transparency, and procedural fairness if it is to rebuild trust in multilateral governance. These signals indicate that commitments will not be arbitrarily revised when alternative political or economic bargains arise. Monitoring, accountability, and dispute resolution mechanisms therefore remain central to sustaining institutional credibility in international agreements. Against this background, efforts to establish a global plastics agreement must address not only regulatory design but also the fundamental objectives that allow commitments to emerge and endure.

“Monitoring, accountability, and dispute resolution mechanisms therefore remain central to sustaining institutional credibility in international agreements.”

## Three lessons for advancing a new plastic treaty

Biosphere processes operate on temporal scales that do not align with electoral cycles or negotiation timelines. Governance architectures that rely on global consensus are therefore vulnerable to short-term political changes. Focusing on underlying commitments can help to address this mismatch. By reinforcing narratives, clarifying normative justifications, aligning individual and joint interests, and strengthening institutional enforceability, governance systems can preserve the long-term attractiveness of cooperative behavior even under conditions of geopolitical fragmentation.

“Biosphere processes operate on temporal scales that do not align with electoral cycles or negotiation timelines.”

First, negotiations may benefit from a more process-oriented logic. Resetting expectations and openly addressing historical grievances could help to renew mutual trust. Institutionalized dialog mechanisms—analogous to truth and reconciliation processes—might allow participants to acknowledge asymmetries, historical responsibilities, and perceived injustices in environmental governance.

Second, the institutional landscape and its underlying narratives warrant closer examination. Understanding how national and international narratives shape environmental policies can help to clarify motivations and interests. Such analysis may also reveal how scientific knowledge is interpreted and mobilized across political contexts, influencing the prospects for collective action.

Third, greater attention should be paid to existing plurilateral and regional initiatives. The EU Single-Use Plastics Directive, regulatory measures adopted by Canada, and similar policies introduced in New South Wales demonstrate that commitments can be made at national and sub-global levels. These initiatives function as policy laboratories and may gradually expand into renewed multilateral cooperation.

Reframing governance around commitments does not guarantee immediate results. Yet, in a period characterized by declining trust, geopolitical instability, and fragmented authority, rebuilding the narrative and institutional foundations of cooperation may become a necessary first step toward a new social contract capable of addressing planetary risks in a multipolar world.

## Supplementary information

Peer Review File

## References

[CR1] Canuto O, Saraiva B. BRICS in times of tectonic shifts. Policy Center For The New South, 2025

[CR2] Dauvergne P, Allan JI (2025) UN politics won’t deliver an ambitious plastics treaty. Science 390(6768):7 41037603 10.1126/science.aec1353

[CR3] Editorial (2025) UN plastics treaty must be ambitious. Nature 643:303 40634748 10.1038/d41586-025-02064-1

[CR4] Johnson SG, Bilovich A, Tuckett D (2023) Conviction narrative theory: a theory of choice under radical uncertainty. Behav Brain Sci 46:e82 10.1017/S0140525X22001157 35634729

[CR5] Landrigan PJ et al (2025) The Lancet countdown on health and plastics. Lancet 406(10507):1044–1062 40769171 10.1016/S0140-6736(25)01447-3

[CR6] Müller GG, De Man P (2025) Needs and deeds of EU external action: adapting to global shifts. Eur Foreign Aff Rev 30(3):327–348

[CR7] North DC (1993) Institutions and credible commitment. J InstitTheor Econ (JITE)/Z für die Gesamt Staatswiss 149:11–23

[CR8] Northrup O. Grounds for commitment. University of California, Santa Cruz, 2014

[CR9] ten Hietbrink S et al (2025) Nanoplastic concentrations across the North Atlantic. Nature 643(8071):412–416 40634739 10.1038/s41586-025-09218-1 PMC12240857

