# Supplementary material for: Navigating the plastic crisis: How Commitments Can Lead to Polycentric Governance
Source: EMBO Rep. 2026 Apr 24;27(10):2522–5. doi: 10.1038/s44319-026-00784-7 (PMC13219513; doi:10.1038/s44319-026-00784-7)
Supplement: Supplementary file 1 — Peer Review File [file 44319_2026_784_MOESM1_ESM.pdf]

EMBO reports

Navigating Plastic Crisis. How Commitments Can Lead to Polycentric Governance

Matthias Braun and Anna-Katharina Hornidge

Corresponding author(s): Matthias Braun (matthias.braun@uni-bonn.de)

Review Timeline:		
	Submission Date:	25th Mar 26
	Accepted:	13th Apr 26

Editor: Holger Breithaupt

Transaction Report:

This article was editorially reviewed
